# Inflammatory myofibroblastic tumor of the lung: A rare endobronchial mass

**DOI:** 10.1016/j.rmcr.2020.101285

**Published:** 2020-11-10

**Authors:** Merian E. Kuipers, Hans Dik, Luuk N.A. Willems, Bart P.C. Hoppe

**Affiliations:** a Leiden University Medical Center, the Netherlands; b Alrijne Hospital Leiderdorp, the Netherlands

**Keywords:** Rigid bronchoscopy, Inflammatory myofibroblastic tumor, Inspiratory wheeze, Respiratory medicine, Tumor pathology

## Abstract

A 42-year old male was referred with a 6-week history of new onset dyspnea. The patient had normal vital signs, no relevant medical history and the only abnormality was a left sided inspiratory wheeze. No abnormalities were seen on the chest X-ray. A bronchoscopy was performed which showed a well-circumscribed hypervasculated mass in the left main bronchus. A biopsy was taken, which was complicated after the procedure by dislocation of the mass and coughed up by the patient. Both samples were send for pathologic review. A contrast CT was performed which showed a localized remaining mass in the left main bronchus and no lymph node involvement. Pathological evaluation showed spindle-shaped cell proliferation with mitotic activity in the second larger tissue which could be consistent with an inflammatory myofibroblastic tumor (IMT), whereas the first biopsy sample only showed granulomatous inflammation. Following multidisciplinary review the diagnosis of IMT was made and a treatment plan was decided. Because of the localized position of the mass the patient was treated with laser coagulation via rigid bronchoscopy instead of surgery. Bronchoscopic review afterwards showed complete resolution of the mass and the dyspnea had resolved. This case highlights the difficulty of making the IMT-diagnosis and the option of treating it with laser coagulation via rigid bronchoscopy.

## 1 Introduction

Inflammatory myofibroblastic tumor (IMT) is a rare type of tumor presenting mostly in children and patients under 40 years of age [1]. It is a neoplasm associated with the anaplastic lymphoma kinase (ALK) gene and classified as a subset of the inflammatory myofibroblastic pseudotumors. The primary locations are the abdomen, pelvis, lung, orbit and retroperitoneum but it may occur anywhere [2]. In the lung it is mainly found parenchymal or pleural, but rarely endobronchial [3]. Of all lung tumors IMTs account for 0.04%–1.0% and may be difficult to diagnose due to a broad differential diagnosis, the rarity of the disease and difficult pathological features [4,5].

## 2 Case report

A 42-year-old non-smoking male presented to the outpatient clinic with a 6 week-history of dyspnea. His medical history was otherwise unremarkable. General examination revealed a healthy male without signs of respiratory distress and normal vital signs. Breath sounds were unremarkable except for a left sided inspiratory wheeze. Blood tests were normal and a chest X-ray revealed no abnormalities.

**Fig. 1:**
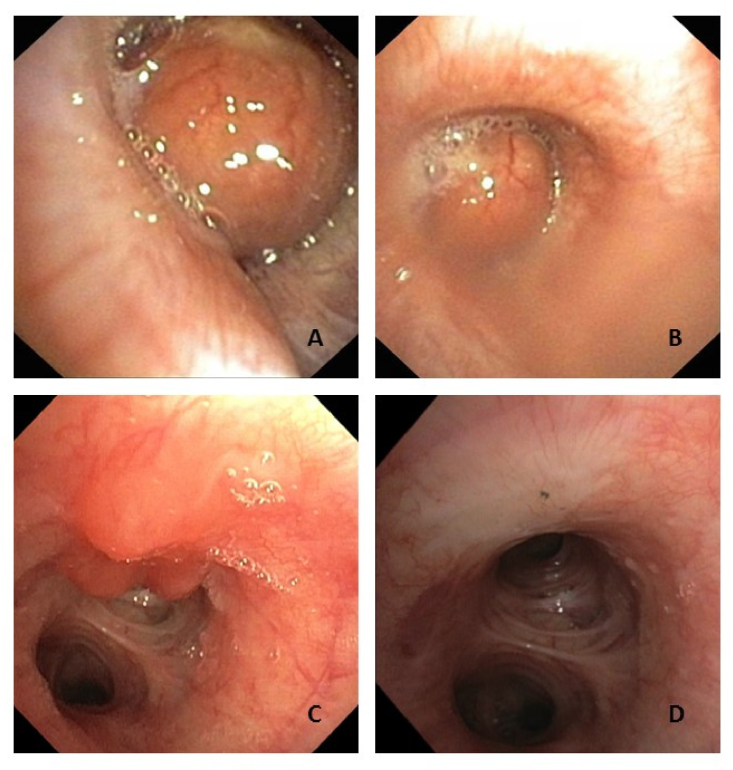
Bronchoscopic images showing A-B; the tumor in the left main bronchus, C; the left main bronchus three weeks after biopsy of the mass and D; the left main bronchus after laser coagulation, showing white scar tissue.

In order to evaluate the inspiratory wheeze a bronchoscopy was performed which showed a well-circumscribed hypervasculated mass on the lateral wall of the left main bronchus with subtotal occlusion of the left main bronchus (Fig. 1A and B). A biopsy was taken for histological examination. However, 1 h after the procedure, the patient coughed up a large piece of tissue which was histologically confirmed to be a large part of the ‘remaining tumor’. This was further confirmed by a second bronchoscopy performed three weeks later showing a relatively small residual tumor (Fig. 1C).

**Fig. 2:**
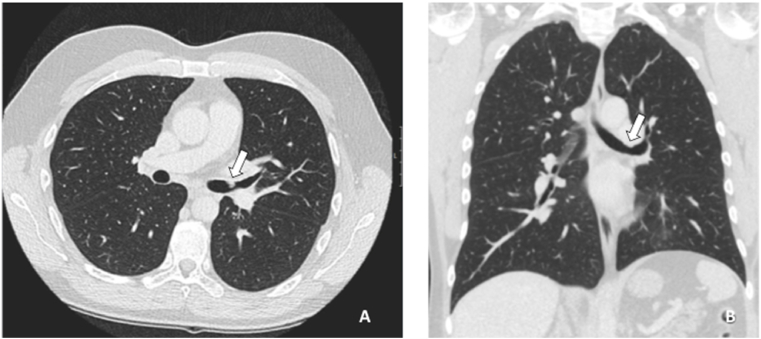
Computed Tomography A; transversal and B; coronal images of the chest after initial bronchoscopy showing a remaining lesion in the left main bronchus (arrow).

A computed tomography scan was performed to assess the lymph nodes and parenchymal involvement which showed a fibrotic lesion in the left main bronchus (Fig. 2).

**Fig. 3:**
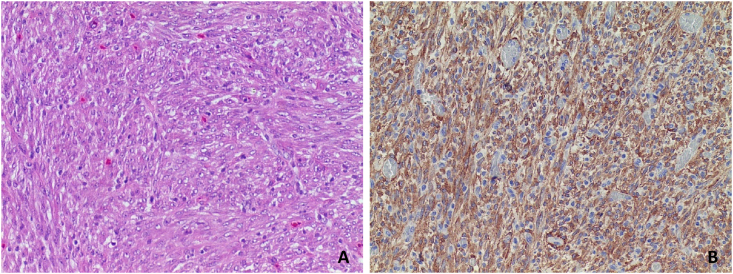
Immunohistochemistry. Image showing A; HE-coloring of lesion rich in spindle cells, B; spindle-cells strongly positive for ALK.

The histological assessment of the biopsy taken during the original bronchoscopy showed a granulomatous inflammation. Meanwhile the immunohistochemistry (Fig. 3) of the dislocated tumor showed an atypical spindle-shaped cell proliferation with mitotic activity and was positive for EML4 (echinoderm microtubule associated protein-like 4) and ALK. These findings were most consistent with a diagnosis of an inflammatory myofibroblastic tumor. The presence of ALK suggests the neoplastic nature of this tumor which makes it a distinctly different entity than an inflammatory pseudotumor (IPT) [6]. The definite diagnosis of IMT was made in a multidisciplinary meeting taken into account the patient characteristics and all pathological features. The agreed management plan was one of laser coagulation and (local) follow-up.

Laser coagulation of the remaining tissue was performed by means of a rigid bronchoscopy to remove the remaining tumor and prevent local recurrence. The end result observed after 3 months was a small area of pale-scarred mucosa (Fig. 1D), which will be followed-up by means of 6-monthly bronchoscopy.

## 3 Discussion

IMT of the lung can present with cough, chest pain, dyspnea or, less typically, hemoptysis. B-symptoms such as fever, weight loss and malaise may also be present. Lab results may show a raised erythrocyte sedimentation rate, microcytic anemia and thrombocytosis [5]. Typically, however, they are asymptomatic and are incidentally discovered on a chest X-ray [7]. Due to the varying nomenclature of IMT the incidence is difficult to report, but estimates range from 0.4% to 1%, making it one of the least common tumors of the lung. The etiology and pathogenesis remain unclear but it has been linked to a post-inflammatory process [8]. [][][].

Histological evaluation of IMTs generally show variably cellular spindle cell proliferation with often a lymphocytic inflammatory infiltrate [5]. Three histopathological patterns are described by Coffin et al.: a compact spindle cell pattern, a vascular/myxoid pattern and a hypocellular fibrous pattern. In this case, the spindle cell pattern was predominant [2]. It is often difficult to make the diagnosis of IMT based on a single biopsy, and complete resection is almost always necessary for a definitive diagnosis. Interestingly, in this case, the dislocation of the tumor most probably resulted in a rapid and complete diagnosis.

IMT are usually considered to be benign lesions, metastasis can occur in 2–5%, but local recurrence can be up to 25%, depending on the location, resectability and multinodularity [5,9]. Complete surgical resection is the treatment of choice for all IMTs, but bronchoscopic resection has been proven to be successful in the case of an endobronchial tumor [10]. In case of metastasis or contra-indications to surgical resection chemotherapy, radiotherapy and corticosteroids can be considered [3].

## 4 Conclusion

This case highlights the difficulty of making the diagnosis of IMT by means of a bronchoscopy with transbronchial biopsy. Often more tissue is needed to make a correct diagnosis. In this case more tissue was obtained ‘accidently’. Metastasis is extremely rare and local treatment with surgery is the treatment of choice. However, as in this case, an endobronchial approach via (rigid) bronchoscopy can be used for radical treatment of the tumor.

## Declaration of competing interest

None.
